# Demodicosis in Different Age Groups and Alternative Treatment Options—A Review

**DOI:** 10.3390/jcm12041649

**Published:** 2023-02-19

**Authors:** Izabela Chudzicka-Strugała, Iwona Gołębiewska, Grzegorz Brudecki, Wael Elamin, Barbara Zwoździak

**Affiliations:** 1 Department of Medical Microbiology, Poznan University of Medical Sciences, Rokietnicka 10, 60-806 Poznan, Poland; 2 Earth and Life Institute (ELI), Université Catholique de Louvain, Croix du Sud 2, 1348 Louvain-La-Neuve, Belgium; 3 Group 42 (Healthcare), Masdar City, Abu Dhabi P.O. Box 112778, United Arab Emirates

**Keywords:** *Demodex* spp., demodicosis, bacterial coinfections, essential oils

## Abstract

Infestation with *Demodex* mites is a common occurrence, especially in adults and the elderly. More recent attention has been paid to the presence of *Demodex* spp. mites in children, even ones without comorbidities. It causes both dermatological and ophthalmological problems. The presence of *Demodex* spp. is often asymptomatic, thus it is suggested to include parasitological investigation tests in dermatological diagnostics, in addition to bacteriological analysis. Literature reports show that *Demodex* spp. are related to the pathogenesis of numerous dermatoses, including rosacea or demodicosis gravis, and common eye pathologies reported by patients such as dry eye syndrome or ocular surface inflammatory conditions, such as blepharitis, chalazia, Meibomian gland dysfunction, and keratitis. Treatment of patients is a challenge and is usually prolonged, therefore it is important to carefully diagnose and properly select the therapy regimen for the treatment to be successful, and with minimal side effects, especially for young patients. Apart from the use of essential oils, research is ongoing for new alternative preparations active against *Demodex* sp. Our review was focused on the analysis of the current literature data on the available agents in the treatment of demodicosis in adults and children.

## 1. Introduction

The human body is colonized by many microorganisms, including bacteria, fungi, and ectoparasites, often related only to the human body. The multicellular, external microorganisms inhabiting the human skin and other mammals include the *Demodex* spp. mites. They are considered to be one of the most common microscopic ectoparasites [1]. The extent of their pathogenic potential is still a current topic of many global discussions, although microorganisms have been known to dermatologists, ophthalmologists, and veterinarians for nearly 180 years. So far, only two species of *Demodex* mites have been described that are associated with human demodicosis: *D. folliculorum* (Simon, 1842) and *D. brevis* (Akbulatova, 1963) [2]. The pathogenic role of *Demodex* spp. in humans is the subject of much debate since these microorganisms can be present in humans without showing any symptoms. It was found that this microorganism occurs in the majority of people over 60 years of age (84% of the population tested) and in all people over 70 (100%) [1]. However, at the same time, *Demodex* spp. play an important role in the development of many skin and eye diseases [3]. In addition, certain factors such as immunosuppression, diabetes mellitus, vasodilatation factors, and/or sebaceous gland hyperplasia may trigger the proliferation of *Demodex* spp. [4]. This early stage of demodicosis is considered a subclinical form with no clinically apparent changes. However, clinical changes are not observed. If the proliferation process is progressing, the so-called primary demodicosis (pityriasis folliculorum) shows whitish, vesicular scales at the base of the hair and diffuse erythema on the skin. Unfortunately, sometimes these symptoms are so discreet or unnoticed by a dermatologist that this disease entity is often underdiagnosed [4]. The immune system response of the infested host is ineffective, resulting in the development of a clinical form of “rosacea-like demodicosis” and other variants of infestation, including ocular demodicosis, *Demodex* folliculitis/abscess, demodectic prurigo, vesicular and follicular eczematidis, and demodectic post-inflammatory pigmentation. However, the exact prevalence of demodicosis in the human population has not yet been estimated [4]. Although it has so far been suggested that these mites do not occur in healthy children under one decade of age [3] or are associated with cases of reduced immunity [5], malignancy, or malnutrition [6], there are more recent data that contradict these reports. In the analysis of Zhang et al., 2021 [7] of over one and a half thousand healthy children aged 3–14 years, *Demodex* spp. mites on eyelashes were found in 12%. Microorganisms were present both in children without eye discomfort and in children with diagnosed eyelash abnormalities (trichiasis, cylindrical dandruff, or scaly discharge at the base of the eyelashes) associated with progressive demodicosis. Moreover, the relationship between living conditions and the higher incidence of *Demodex* spp. in children from rural areas was emphasized [7]. Since *Demodex* spp. mites can also occur in humans as saprophytes, a therapeutic problem also arises. The search for the most effective treatment has been ongoing for years. In the case of ocular demodicosis, standard eyelid hygiene was used, sometimes with sulfur ointment, yellow mercury ointment, pilocarpine gel or local antibiotics, and anthelmintics. Therefore, there are attempts to develop alternative therapies that will be simultaneously effective and their potential for side effects minimized, e.g., with topical treatment based on tea tree oil (TTO) and terpinen-4-ol (T4O) [8]. New, promising possibilities of anti-demodicosis therapies provide essential oils with a democidal effect, but also greater skin tolerance and the possibility of eye demodicosis therapy [9], which is of particular importance especially when the therapy is implemented in pediatric and immunocompromised patients.

### Demodex spp.

*Demodex* spp. belong to a large group of mites with a very wide geographical range, covering many climatic zones. Currently, over 100 species are known, and the most significant species for humans are *D. folliculorum* and *D. brevis*. They are highly specialized parasites closely connected to their host, attacking different skin areas of one host (Lacey et al., 2009) [10]. The presence of this parasite is found in almost all parts of the human body (including the nose area, forehead, chin, cheeks, nasolabial furrow, hair follicles, around the mouth, eyes, breast nipples, and genital area) where hair or lash roots and sebaceous glands are present. The place of their occurrence is related to the appropriate living and reproduction of these parasites, also in terms of the temperature needed for their development.

*Demodex* spp. mites have a worm-like shape, and their surface is covered with a thin cuticle. Elongated *D. folliculorum* can be 0.3 to 0.4 mm long. Its presence is found in the outlets of hair follicles, in which it forms clusters composed of several individuals. Shorter and spindly in its shape, *D. brevis* reaches a length of 0.2–0.3 mm and also has shorter legs. It usually attacks alone and locates itself deep in the apocrine and sebaceous glands of the face, chest, Meibomian gland (causing its destruction), in the external auditory canal (causing periodic leakage and unpleasant odor from the ears), or in the genital area [11,12].

**Figure 1:**
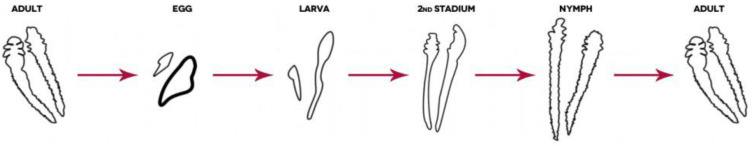
Life cycle of the *Demodex* mite.

The life cycle of the parasite (Figure 1) is approximately 3 weeks (14–18/24 days) and the adult mites live for approximately one week. Parasites feed primarily on blood and blood plasma filtrate, blood, the secretion of sebaceous glands, and epithelial cells. The female lays 20–24 eggs, and their size reaches 50–60 μm, from which the larvae hatch, morphing into nymphs (protonymph to deutonymph) and further into adults, which avoid sunlight and actively forage at night [3].

The anterior body of *Demodex* has a gnathosoma with mouthparts, while the rest of the body is made up of podosome and opisthosoma. The *Demodex folliculorum* has chelicerae located on the gnathosoma, which are sharp, dagger-like, and more developed than in *Demodex brevis*. The function of chelicerae is to absorb and grind food, while legs are supported by pedipalps. Both *Demodex* species have four pairs of legs on the podosome [13]. It is estimated that parasites move at a speed of approximately 16 mm/h [3,14]. The mouths of adults are as sharp as a needle, thanks to which the parasite penetrates the host’s cell directly and absorbs nutrients from it. The parasite’s jaw secretes lytic enzymes to pre-digest the food, after which the dissolved nutrients are sucked into its gastrointestinal tract [3].

*Demodex* spp. mites prefer moist and warm environments. Humans become infected with the parasite that penetrates healthy skin and looks for sebaceous glands in hair follicles rich in sebum. Ocular signs may include eyelash debris such as waxy debris, scaly debris, cylindrical dandruff, or erythema and telangiectasia of the eyelid [15,16].

## 2. Literature Search Strategy

This review presents the anatomical features of *Demodex* sp., the life cycle, the mode of transmission, and its pathogenicity and coinfections in different age groups. Risk factors for infestations and accompanying symptoms, as well as prophylaxis, were taken into account. An analysis of routinely used diagnostic methods and available alternative therapeutic options was undertaken. Although there are more and more reports on this microorganism, opinions on its pathogenicity still vary. Therefore, the primary goal of our article was to review the literature reports on the currently used and alternative treatment options for *Demodex* sp. infestations, as well as the available, particularly routine, diagnostic methods in the diagnosis of demodicosis. A literature search was performed using PubMed and the Cochrane Library. Articles on the potential therapeutic possibilities of demodicosis have been limited to the years 1980–2022. The search covered original studies, guidelines, and syntheses. We also included some in vitro analyses of alternative preparations based on essential oils that can become helpful and effective in the fight against this microorganism. The analyses varied in both quality and design, some of which were randomized controlled trials.

## 3. Demodicosis Etiology and Coinfections

It is believed that the effect of *Demodex* sp. mites on humans causes direct damage. It leads to distension of the hair follicles, microabrasion, epithelial hyperplasia, and reactive hyperkeratinization (*D. folliculorum*), as well as mechanical blockage of the Meibomian glands and granulomatous reactions due to the presence of a chitinous skeleton (*D. brevis*). There are many factors involved in hair loss from *Demodex* spp. infestations and the process likely needs more research. *Demodex* spp. produce an immunologically active lipase that stimulates inflammation [17]. In the hair follicles, lipases produced by *Demodex* spp. can lead to degradation of the follicle epithelium, and consequently, increased invasion causes excessive destruction of the hair follicles and perifollicular inflammation [18]. In addition, the opening of the vesicle may also be mechanically blocked by *Demodex* spp., and extra-alveolar mites may induce a granulomatous reaction to the chitinous skeleton [19]. An example is inflammation and the suggested involvement of mites in androgenetic alopecia (AGA), which is limited to the area around the sebaceous glands and infundibulum. As a result of the activation of T lymphocytes infiltrating the hair follicles, a process occurs in which the hair follicle becomes fibrotic. These patients are more predisposed to infestation by producing more oils in the hair follicles. *Demodex* spp. feed on sebum and keratinocyte debris [20], and although they are not the direct cause of AGA, prolonged infestation by the parasite causes the hair cycle from anagen to telogen and follicle depletion [21]. Although many aspects of dissecting folliculitis of the scalp, associated with patchy hair loss [22], are yet to be elucidated, an inflammatory reaction is suggested due to the presence of microorganisms such as *Demodex* spp. and associated bacteria (e.g., Propionibacterium (Cutibacterium) acnes, Staphylococcus aureus) that are present in the hair follicles, and they induce these processes [21]. It is indicated that in patients with rosacea, there is an increase in folliculitis associated with the presence of *Demodex* spp., which correlates with a decrease in type 2 cytokines (IL-2). Research by Ricardo-Gonzalez et al., 2022 [20] in animal models showed the key role of the so-called immune checkpoint, which includes, coupled with the hair growth cycle dermal ILC2 (innate lymphoid cells) and IL-13, maintaining skin integrity and reducing pathological *Demodex* spp. mite infestation. It was observed that as a result of their dysfunction and the loss of such a barrier, colonization of *Demodex* spp. resulted in increased epithelial proliferation and, through the current inflammation, genetic changes in repair programs, which consequently led to the depletion of the hair follicles [20]. Moreover, *Demodex* spp. acts as a vector for bacteria such as *Streptococci*, *Staphylococci* (on their surface), and *Bacillus oleronius* (in the abdominal cavity). They are believed to be a potential cause of chalazion and MGD. Additionally, *Demodex* spp. can induce an inflammatory response in the host through delayed hypersensitivity or an innate immune response of the host, due to the action of proteins present inside the mite, their debris, or waste [23].

### 3.1. Immunological Response

*Demodex* spp. crawl, multiply, and excrete feces, and their entire life cycle takes place in the sebaceous gland. Dead individuals decompose into sebum and, together with the remaining feces, lead to mechanical and chemical irritation of the skin and hypersensitivity reactions. In addition, they trigger an immune response in the form of cellular activity with the participation of T lymphocytes and an increase in the production of pro-inflammatory cytokines, primarily IL-17. Kim et al. suggested that in the tears of patients with eyelid margin inflammation and ocular surface due to a *Demodex* sp. infestation, the concentration of IL-17 is higher in comparison to the people without *Demodex* spp. infestation [24]. Zhang et al., 2018 [25] have shown that infestation with *Demodex folliculorum* activates IL-17/MMP-9 signaling and thus may even enhance the function of the corneal epithelial barrier [25]. Il-17 causes inflammation and blockage of the glands and damage to the eye surface [26]. Moreover, the secretion of the vascular endothelial growth factor is stimulated, leading to a vascular reaction. Within a short time, inflammation symptoms develop as itching, pain, redness (red spots), papules, pimples, acne, or sties [27]. During the *Demodex* spp. infestation of a human host, the immune system response is stimulated with the participation of Toll-like receptors (TLR) [28]. It is suggested that the pro-inflammatory response of keratinocytes via TLR-2 is also induced by a component of the *Demodex* spp. exoskeleton, i.e., chitin [29]. The enzymes secreted by the mites include proteases. The skin surface is covered with serum deposits of the IgD immunoglobulin and two inhibitors of serum proteases, alpha-1-trypsin and alpha-1-antichymotrypsin, causing a specific immune response in the human body (Tsutsumi, 2004) [30]. Lacey et al. suggested that *Demodex* spp. are capable of secreting bioactive molecules, influencing the immune reactivity of sebaceous cells, and therefore they can modulate the TLR signaling pathway of the immortalized human sebocyte lineage. It was shown that the increase in the number of *Demodex* spp. influenced the secretion of interleukin-8 by these cells [31].

### 3.2. *Demodex* spp. and Coinfections

*Demodex* spp. infection is known for its coincidence with bacterial and fungal infections, as it can facilitate the transmission of other microbes to adjacent tissues or other people [32]. Examples of co-infecting organisms and associated manifestations are summarized in Table 1.

The studies of Liang et al., 2021 [38] emphasize the participation of other microorganisms in eye infestations, especially bacteria, classifying them as co-pathogens of *Demodex* sp. such as *Acinetobacter calcoaceticus*, *Novosphingobium*, and the *Anoxybacillus* and *Pseudomonas* genera [38]. It has been shown that in patients with demodicosis, the combination of anti-degenerative and antibacterial therapy often gave better results [39]. Moreover, it is indicated based on the clinical observations of Hung et al., 2021 [40] on coexisting infestation with *Demodex* sp. infestation in patients with herpetic keratitis [40]. Due to the possibility of transmitting pathogenic microorganisms, inflammatory reactions may develop even after the elimination of demodicosis. In patients with *Demodex* spp. papulopustular rosacea or ocular rosacea, stimulation of the inflammatory response is observed due to the antigenic proteins and stimulation of cathelicidin production of *B. oleronius* [41], bacteria that have been isolated from the parasites of *Demodex* spp. [10]. In addition, the participation of Bacillus proteins in the increase in the ability to migrate, degranulate, and produce cytokines by neutrophils in rosacea in the course of inflammatory erythema is indicated [42,43]. These proteins can also lead to abnormal wound healing, which has been shown in patients with rosacea and the development of corneal ulcers [42]. Moreover, attention is also drawn to the participation of other bacteria in the development of pustular and ocular rosacea, such as *S. epidermidis* [44] or *S. aureus* with pathogenic potential [35]. In the case of patients with rosacea and Staphylococcus analysis [45], the dependence of the variety and amount of proteins secreted by isolated bacteria on temperature was demonstrated. Patients with rosacea have a higher facial temperature than those without rosacea, leading to an increase in the variety and quantity of these proteins [45]. Therefore, it is suggested that skin with rosacea has an altered microenvironment and alters the skin microbiome, leading to exacerbation of symptoms [46]. The action of, for example, *S. epidermidis* is modified, leading to its participation in skin inflammation, as the antigens of this bacterium are recognized by TLR-2 [41]. Therefore, taking into account co-infections, it is worth considering the use of an extended therapeutic spectrum in the treatment of demodicosis [47]. 

## 4. Incidence of Demodicosis in Different Patient Groups

### 4.1. Demodicosis in Children and Teenagers

It is now believed that children under 16 are relatively rarely affected by *Demodex* spp. Some studies indicate a 13% share of *Demodex* spp. infestations between the ages of 3 and 15 years, and at the age of 19–25, the percentage increases to 34% [48]. In a study by Zhang et al., 2021 [7] on eyelashes in a group of nearly 1600 healthy children aged 3 to 14 years, a 12% prevalence of *Demodex* spp. was demonstrated, with *Demodex folliculorum* dominance. The higher detection rate of *Demodex* spp. was correlated with the autumn–winter period. Children with *Demodex* spp. infection usually did not report any discomfort. In the subgroup aged 3–6, a higher percentage of infections was found in children from rural areas. On the other hand, in the subgroup of older children aged 7–14, the symptoms of demodicosis were found more often, e.g., abnormalities of the eyelashes, cylindrical dandruff, or a scaly discharge at the root of the eyelashes [7]. 

It is believed that even close contact with the newborn in the first days of life or contact with the mother’s skin during breastfeeding is associated with the transmission of *Demodex* sp., but due to the low production of sebum, the density of this microorganism is low [49]. Moreover, even in the presence of eyelid inflammation or conjunctivitis in pediatric patients without comorbidities, many physicians/ophthalmologists believe the symptoms are not related to a potential *Demodex* spp. infestation. Dermatological demodicosis develops in young patients with primary or secondary immune suppression due to disease or drugs [50,51]. In immunocompromised pediatric patients, e.g., leukemia, *Demodex* spp. infestation may lead to severe inflammation of the face and eyelids [52]. In the studies of Kaya et al., 2013 [6] in immunocompromised children, apart from malignancy, another cause of immunosuppression, namely, malnutrition, was also highlighted as a risk factor for *Demodex* spp. infestation. Studies have shown that *Demodex* spp. is involved in infestations of up to one-quarter of malnourished children and one-third of children with cancer [6]. Not all aspects of the pathogenesis and immune responses in *Demodex* spp. infestation, especially in children, are known. An example is the case of a 7-year-old immunocompetent girl described by Guerrero-Gonzalez et al., 2014 [53]. Long-term use of topical steroids had resulted in local immunosuppression and secondary development of severe crusted demodicosis [53]. There are more recent data showing support for considering *Demodex* sp. as an etiological agent in the development of eyelid inflammation and conjunctivitis in pediatric patients without reduced immunity, even in the first decade of life [23]. In children, demodicosis is not commonly reported, therefore *Demodex* spp. infestation in the pediatric population should be of particular concern. In eyelash studies by Huang et al., 2022 [54] on 446 children with chalazia aged 7 months to 13 years, it was shown that *Demodex* spp. infestation occurred significantly more often (almost 53%) compared to the control group of children with non-inflammatory eye diseases (0%). Chalazia is considered a common pediatric problem due to its recurrence, higher frequency in children than in adults at a level of up to 25% [33], poor coordination of treatment, and difficulty in prevention. Hence, the important role of *Demodex* spp. as a risk factor for the development of chalazia in children is emphasized. In addition, special attention should be paid to preventive check-ups and comprehensive treatment in the case of an infestation in order to prevent the recurrence of a chalazion in children [54]. Xiao et al., 2022 [55] also showed a significant correlation between demodicosis and childhood chalazia. In addition, the incidence and amount of *Demodex* spp. were found to influence its severity [55]. The importance of *Demodex* spp. infestation in the pediatric population is also indicated by studies by Liang et al., 2010 [33] in patients aged 2.5 to 11 years with chronic blepharitis and conjunctivitis, in which conventional treatments were observed ineffective. The study showed the presence of *Demodex* spp. in all analyzed patients, and the implemented treatment with TTO (tea tree oil) resulted in the resolution of eye irritation and inflammation and a reduction in the number of *Demodex* spp. Hence, even in the case of children without accompanying immunological disorders, it is worth considering demodicosis in blepharitis and conjunctivitis, especially in the case of therapeutic failures with conventional methods [33]. 

### 4.2. Demodicosis in Adults and Elderly

It is estimated that the incidence of *Demodex* sp. increases with age, hence in middle-aged and older adults, it reaches 100%. Moreover, it reaches 84% of the total population over 60 and 100% of the total population over 70 [56]. It is estimated that the density of *Demodex* sp. in the general population is less than <5 *Demodex* sp./cm^2^. The average rate of infestation with *Demodex* spp. in the world population is estimated at approximately 60%. It can occur in the skin of people without any symptoms, and the infestation rate does not equal its incidence [23]. The incidence depends on the number of *Demodex* spp. individuals on the human skin and the condition of the immune system [57,58]. However, the infestation is considered to develop when there is an increase in the number of mites in the sebaceous unit or its penetration into the dermis [49]. Spreading of *Demodex* spp. in humans occurs primarily through direct contact with an infected person, e.g., contact with the skin of the face, kisses, shaking hands, using shared towels, bed linen, pillows, blankets, cosmetics (including lipstick, lip gloss, creams, mascaras, powders, powder sponges, and eyeshadows), and hairbrushes at home, at a hotel, or at a hairdresser or beauty salon. *Demodex* spp., as well as other mites, can also spread through used clothes (e.g., second-hand clothes stores, sharing clothes) [48]. In studies by Sedzikowska et al., 2021 [59], it has been also shown that sharing facial cosmetics (e.g., mascara and lipsticks) can be a source of transmission of *Demodex* sp. among users [59]. Studies by Vargas-Arzola et al., 2020 [60] in a university population of over 8000 subjects showed a negative correlation with age. The highest incidence was found in young adults (19–22 years old), and the involvement of cosmetics, face creams, eyeliner, glasses, or contact lenses in infestation with *Demodex* spp. was highlighted [60]. In a 10-year observational study by Biernat et al., 2018 [61] of patients aged 17–88 with blepharitis, a *Demodex* spp. infestation was shown in 62% of people. The study found an increase in the incidence of *Demodex* spp. in patients over 50 years of age and in those who reported itchy eyes [61]. Research by Cheng et al., 2021 [27] in patients (mean age of the study population 65.7 ± 12.5 years) with dry eye syndrome, where the main reported symptom was ocular pruritus suggesting the involvement of non-histaminergic itching pathways and cylindrical dandruff, showed a 69% incidence of *Demodex* spp. The analyses highlight the importance of such symptoms as helpful in determining the *Demodex* spp. etiology. Furthermore, it has been shown that the eye pathology of *Demodex* spp. etiology is attributed to individuals in the presence of mite numbers ≥ 2 [27]. Therefore, it is worth paying attention to the course of the disease and the ineffective traditional therapies used and taking into account the potential possibility of *Demodex* spp. as the etiological agent. Further under consideration is the potential use of corticosteroids in demodicosis or in the case of unrecognized demodicosis, which would alleviate inflammation and granulomatous reactions but, by impairing immunity, result in delayed clearance of the mites from the host’s body [27]. As *Demodex* spp. proliferation leads to demodicosis development, it may present a wide variety of non-specific symptoms, e.g., unexplained itching, dry and blotchy facial skin, hypersensitivity, or non-specific papulopustular and nodular lesions, while also imitating other dermatoses [18,62]. Although *Demodex* sp. infestation is considered much more common in the elderly, the role of the patient’s age has not been fully explained, especially in ocular demodicosis. The study by Li et al., 2021 [56] showed higher *D. brevis* infestation, more severe corneal lesions, and MGL in young patients with demodicosis of the eye (<35 years of age) than in older patients (>45 years of age) with *D. folliculorum* dominance. In the study, differences in the symptoms presented by the patients were observed. For the elderly, eye dryness, eye fatigue, itching, and MGD (Meibomian gland dysfunction), which led to ocular surface discomfort, predominate. In younger patients, however, blurred and disturbed vision, eye pain, severe MGL (Meibomian gland loss), and corneal lesions were reported [56]. Hence, also in the case of the presence of *Demodex* sp. in ocular inflammation and blepharitis, it is believed that the increase in the number of *Demodex* sp. mites causes or worsens symptoms, although the minimum number necessary to induce symptoms has not been demonstrated so far [56]. Many scientific studies give ambiguous results in the context of the correlation of demodicosis with age. Therefore, the association of demodicosis with only the aspect of age cannot be a separate exponent in the diagnosis of *Demodex* spp. infestation, and the contribution of age requires further analysis. Therefore, regardless of the patient’s age, appropriate diagnostics for *Demodex* spp. should be performed, if there are grounds for this.

## 5. Demodicosis—Predisposing Factors and Clinical Presentation

*Demodex* spp. has been considered the etiological factor of rosacea, chronic inflammatory dermatosis of the facial skin since the 1930s. Symptoms of facial dermatosis are manifested by the presence of many small, domed erythematous and maculopapular papules that arise against the background of permanent inflammatory erythema. It has been suggested that an increase in mite density per cm^2^ (>5) correlates with the pathogenic potential. Therefore, there is also a seasonal exacerbation of rosacea in the warmer spring and summer months, associated with the increase in the number of *Demodex* spp. Temperatures in the range of 16 °C to 20 °C have been found optimal for the development of *Demodex* spp. Additionally, it has been observed that temperatures below 0 °C and above 45 °C have a destructive effect on these parasites, and a temperature of 54 °C is lethal [63].

Skin diseases caused by *Demodex* spp. also include follicular dandruff (associated with excessive use of make-up), perioral dermatitis (as a rash), scabies-like lesions, hairless scalp eruptions, *Demodex folliculitis*, demodicosis gravis (with skin granulomas), and sometimes even association with basal cell carcinoma. In the case of basal cell carcinoma (BCC), apart from UV radiation as the main cause, local factors, e.g., inflammation, irritation, or chronic trauma, are emphasized. Hence, the role of *Demodex* spp. in the etiopathogenesis of BCC is suggested, due to the main location of skin areas colonized by these mites, e.g., around the nose and eye sockets, most often affected by BCC and association with chronic inflammation. Therefore, due to the irritating/traumatic effect inducing chronic inflammation, the importance of demodicosis as one of the factors stimulating carcinogenesis in BCC of the eyelids in predisposed people is also emphasized [64]. Furthermore, the analyses of Sun et al., 2005 [65] showed that cases of basal cell carcinoma were characterized by a high infestation rate among the analyzed cancers [65]. 

In addition, the relationship between the development of demodicosis in patients with a reduced state of the immune system, e.g., in connection with the use of steroid drugs or immunosuppressive preparations, and in the course of leukemia or HIV infection, is emphasized [3]. 

**Figure 2:**
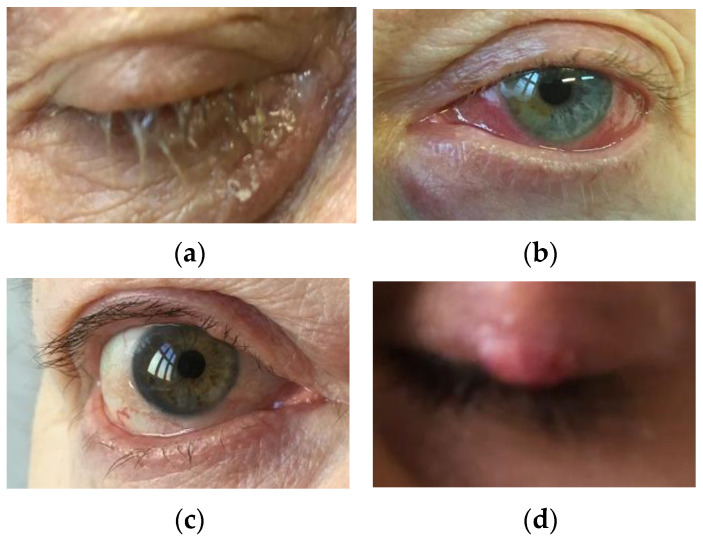
Ocular and blepharal manifestations of demodicosis in adults (authors’ own archive): (**a**) Patient with scales on the eyelashes, eyelid changes (dry, reddened skin lesion); (**b**) patient with conjunctivitis and slight swelling of the upper and lower eyelids; (**c**) patient with conjunctivitis, falling eyelashes (eyelash loss) on the lower eyelid and the beginning of abnormal growth of eyelashes on the upper eyelid; (**d**) patient with a chalazion in the course of demodicosis.

**Figure 3:**
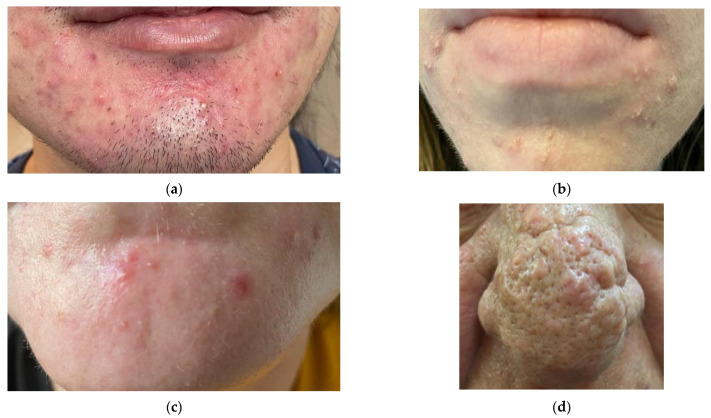
Dermatological manifestations of demodicosis in adults (authors’ own archive): (**a**) Patient with acute skin lesions (rosacea); (**b**) patient with cutaneous demodicosis and bacterial co-infection (*S. aureus*); (**c**) patient with single skin lesions; (**d**) patient with typical skin lesions caused by *Demodex* spp. on the nose, so-called “Demodex nose”.

Due to the possibility of the spread of *Demodex* mites from the skin of the face to the surface of the eyelids, many eye diseases have been observed. The following species are dominant: *D. folliculorum* (especially in the hair follicles) and *D. brevis* (deep in the Zeiss’ glands—sebaceous glands of the eyelashes and Meibomian glands). The presence of these microorganisms is not necessarily related to the disease state, as they can sometimes also appear in small numbers in asymptomatic individuals [3]. Some scientific papers suggest that because *Demodex* spp. mites can be isolated in people who do not present symptoms of dermatological diseases, they constitute a conditionally pathogenic microbiome of the facial skin [66]. On the skin of most people, the presence of *Demodex* spp. mites does not have to lead to symptoms such as acne vulgaris, rosacea, seborrheic dermatitis, or eyelid inflammation [10,67], and they are identified in normal skin [68]. Moreover, to fend off external parasites, humans show a certain type of adaptation: Skin sensory mechanisms, mechanisms of generating itching sensation, and through the use of the care procedure [69]. However, sometimes these mechanisms fail, leading to the development of serious diseases, including rosacea, demodicosis, and blepharitis. Although blepharitis does not pose a direct threat to vision, it can cause keratopathy, neovascularization, and ulceration of the cornea, and permanent changes in eyelid morphology if not properly treated. Therefore, current research largely focuses on the participation in the pathogenesis of the so-called “persistent ectoparasites” such as *Demodex* spp. [70]. Figure 2 and Figure 3 show some common manifestations of demodicosis in adult patients.

Clinical symptoms may also depend on the species of the *Demodex* mites. In the case of the skin, the development of an infestation with *Demodex* spp. is initially characterized by small flaky lumps on the skin of the face, as well as a feeling of dryness and itching. The skin lesions of the face are most often located on the cheeks, paranasal areas, and eyelids. *Demodex* spp., primarily *D. folliculorum*, is the etiological factor of demodicosis (rosacea-like demodicodis), recognized as a disease since 1961. The existence of *Demodex* spp. is more often observed on the skin of patients with rosacea [71]. It is suggested that weight, fatty foods, and high diastolic blood pressure affect the more frequent occurrence of the parasite [72]. Moreover, some researchers suggested that demodicosis is more common in women and they are attacked by *Demodex* sp. easier than men [73]. *Demodex folliculorum* causes skin erythema, peeling, and dryness [74]. Changes known as pityriasis folliculorum appear initially. At this moment, the number of mites is estimated at approximately 60/cm^2^. In the course of rosacea, another characteristic symptom may be the maculopapular rash, and in this case, the number of mites is estimated at 36/cm^2^ [75,76,77]. 

The state of the immune system plays an important role. Additional factors predisposed to increased human colonization by *Demodex* spp. include exposure to sunlight, temperature changes, skin phototype, stress, and using stimulants, e.g., alcohol and tobacco [1]. The course of demodicosis can be both primary and secondary [78]. Both *Demodex* species *D. folliculorum* and *D. brevis* can lead to eyelid margin inflammation. *Demodex folliculorum* primarily causes the so-called “anterior blepharitis”, which is manifested by the characteristic keratin-fatty cuff at the base of the eyelashes. In addition, numerous telangiectasias are observed covering the edges of the eyelids with persistent itching, burning and redness, dryness, foreign body sensation and unusual visual disturbances, brittle eyelashes, and irritation leading to damage to the eye structures, e.g., the cornea. Eyelash growth may be impaired and eyelash loss may be observed, in addition to corneal lesions, Meibomian gland dysfunction (mainly in the case of *Demodex brevis*), and peripheral cloudiness [79]. In the case of an infestation with *Demodex brevis*, the so-called “posterior blepharitis” and symmetrical maculopapular eruptions are observed [1]. The consequence of changes caused by an infestation with these parasites may be an allergic reaction to their chitinous shell and inflammation associated with the secretion of cytokines. Scales are formed on the eyelashes as a result of the peeling of the epithelium, which is associated with the occurrence of rosacea [79,80,81]. The cause of Meibomian gland dysfunction (MGD), apart from the participation of parasites (*Demodex folliculorum* and *Demodex brevis*), is also an infection/colonization with bacteria (including *Staphylococcus aureus*, *Corynebacterium* spp., and *Bacillus oleronius*) and fungi. MGD is caused by blocking the gland orifices and, in consequence, blocking the outflow of the secretions with the participation of, among others, *Demodex brevis*. A granulation reaction occurs, and a link between *Demodex* sp. and the formation of recurrent chalazions and styes has been suggested. Other changes related to the *Demodex* spp. infestation include, among others, dry eye syndrome, ulcerations, keratitis, and endophthalmitis. Recent literature reports the presence of the *Demodex* mite as one of the etiological factors in the development of basal cell carcinoma of the eyelids [23,64,82]. The most common symptoms of demodicosis are summarized in Table 2.

## 6. Diagnosis

**Figure 4:**
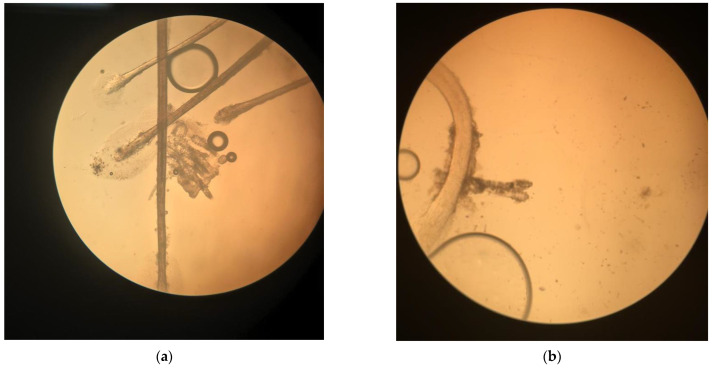
*Demodex folliculorum* (**a**) and *D. brevis* (**b**) under a light microscope, magnification 100×, authors’ samples.

In the diagnosis of demodicosis, the most common method of *Demodex* spp. detection in a patient’s biological material is based primarily on microscopic examination. Samples taken from the patient’s skin, hair, eyebrows, or eyelashes are used. The skin/epidermis samples are obtained from a surface biopsy using cyanoacrylate adhesive glue, skin biopsy, skin scrapings, or using adhesive tape. Eyelashes, eyebrows, hair, or pubic hair are collected via depilation/epilation [89,90]. Other diagnostic methods are also available, although less frequently used, such as dermoscopy and PCR (polymerase chain reaction) [62,87], and the method of confocal laser scanning microscopy in vivo. The advantage of in vivo confocal scanning is the elimination of the need for preliminary preparation for the analysis and possibility of species detection and identification based on the size of *Demodex* spp.: *D. brevis* from 100 to 200 μm and *D. folliculorum* from 200 to 400 μm, at the level of the spinous layer of the epidermis [66,88]. The material collected from the patient is placed on a glass slide in a drop of 10% KOH solution, covered with a coverslip. The sample is analyzed through wet direct preparation. The sample prepared this way is viewed under a light microscope at a magnification of 40× or 100×, mite size is approximately 200 μm (Figure 4) [91,92]. Skin surface biopsy using an adhesive to pull the top layers of epidermis and hairs (with roots) is also used for the detection of *Demodex* spp. Superficial skin scraping (SSS) and tape imprint (TI), which are also applied to detect other parasites, can also be used as diagnostic tools [93]. At times, the skin surface standardization biopsy method (SSSB) is used, which consists of determining the density of parasites per 1 cm^2^ and allows the identification of living, moving parasites of *Demodex* spp. The analysis is performed using a microscope slide with a designated 1 cm^2^ spot containing a drop of cyanoacrylate glue placed on the skin in the affected area. Then, after approximately 30 s, the glass is removed and the material is covered with an immersion oil and a coverslip, and the analysis is performed under an immersion microscope [75,89,93]. Additionally, biological material in the form of scrapings can be subjected to molecular biology methods, e.g., PCR, but, to date, it has not been a standard technique [94].

## 7. Treatment

In the case of a *Demodex* spp. infestation and confirmed demodicosis, the therapy is very complex. It is usually a difficult and lengthy process, lasting up to several months. The choice of drugs is an individual matter. Without an accurate diagnosis and effective treatment to eliminate *Demodex* mites from infected areas, microorganisms can continue to reproduce, causing recurring inflammation, the formation of uneven scars, dilated pores, dilated capillaries, extensive swelling, and pustules [95]. The goal of treatment is the inhibition of the reproduction of the parasites, elimination of the mites, and stopping the recurrence of infestation [83]. The important aspect of preventing the infestation is practicing appropriate hygiene with the use of washing and cleaning products, e.g., soaps, shampoos, and wipes for daily care of the eye and face area. The elimination of the parasite is also promoted by frequent washing of the linen, especially at high temperatures [63]. One of the most important elements of the prophylaxis of demodicosis is the education of patients, but also owners of, for example, beauty salons, in the field of proper hygiene [64]. The systemic treatment of demodicosis is based on the use of antibiotics: Tetracycline, doxycycline, metronidazole, and ivermectin. The most common treatments for demodecosis are metronidazole, but also permethrin, benzoyl benzoate, crotamiton, lindane, and sulfur. The effectiveness of *Demodex* spp. density reduction in the application of metronidazole systemic therapy, even in a short cycle, is indicated. The effectiveness of local therapy with permethrin, as well as crotamiton and benzyl benzoate, has also been demonstrated. However, the use of such agents may be associated with skin irritation in patients. Moreover, there are no unequivocal results of studies that standardize the treatment regimen and indicate its long-term effectiveness [96].

In addition, apart from antibiotics, various medicinal oils are used in therapy including camphor oil, bergamot oil, tea tree oil, peppermint oil, and salvia oil, as well as sulfur ointment, yellow or white mercury ointment, and choline esterase inhibitors [97,98]. Additionally, infrared irradiation or specialized heating glasses are used [99]. Sunbathing and washing the face with warm water were also found to alleviate the symptoms of demodicosis [83]. The area around the eyes can be washed with herbal and plant extracts with antiparasitic activity, e.g., extracts from calamus, celandine, or mugwort [99]. 

Some of the most commonly applied therapies, both conventional and alternative, have been summarized in Table 3.

### 7.1. Antibiotics

Ivermectin works by combating *Demodex* mites externally and internally. Ivermectin is used alone or in combination with metronidazole, which leads to a higher effectiveness of the therapy in the treatment of ocular and skin lesions of *Demodex* spp. [100]. Metronidazole is an antibiotic used in anaerobic bacterial or parasitic infestations, in various combinations and in the form of tablets, ointments, and gels. Its action leads to the destruction of the DNA structure of the pathogen [101]. In the case of MGD, the treatment is to increase the flow of Meibomian secretions by increasing the melting point of the secretions. Therefore, it is recommended to use warm compresses on the affected areas and practice increased hygiene around the eyes [102]. 

### 7.2. Essential Oils

An alternative or complementary form of demodicosis therapy is the use of preparations of natural plant origin in the form of essential oils (EO). They are even more than 80% of a mixture of volatile compounds from the group of (a) terpenes and terpenoids and (b) aromatic and aliphatic components. Due to their antimicrobial and anti-inflammatory properties, they have found use in many therapies, although dosing must be strictly controlled [103]. Research by Liu et al., 2015 [104] showed the activity of clove oil against *Demodex* spp. [104]. 

Long-term treatment of demodicosis often has a short-term effect, therefore the prevention of excessive multiplication of *Demodex* spp. mites also plays an important role, e.g., cleansing the face twice a day with soap-free make-up remover, avoiding mineral oil-based preparations and oily makeup, and periodic exfoliation of dead skin [21]. Skin hygiene based on the use of soap and water does not reduce the intensity of *Demodex* spp. occurrence. Therefore, preparations are sought to counteract the invasion and kill mites. Hence, essential oils or their active compounds, e.g., terpenes, provide great opportunities, in various forms, e.g., lotions, shampoos, ophthalmic liquids, gels, or ointments.

1. Tea tree oil exhibits strong antiseptic, antibacterial, antifungal, antiviral, and antiparasitic properties. It has been found to be effective against aerobic and anaerobic bacteria, molds, and dermatophytes. It has anti-inflammatory and regenerative properties, accelerates the renewal of the epidermis and the process of wound healing, and has the potential to heal ulcers. It supports the treatment of acne, eczema, lichen, and redness, especially in the course of demodicosis. Treatment (lasting approximately 4–6 weeks) using 50% of tea tree oil for intensive massages of the eyelids and daily hygiene and using a shampoo with tea tree extract has been shown to be very effective. It is intended for external use, although it is used in the form of aromatherapy or inhalation. It can also be used as an antitoxin against Australian spider venom. Safe application time is between 1 and 2 days in concentrations of 1–10%, and it can be applied for up to 6 months. In the case of demodicosis, the action of tea tree oil causes the expansion of mites to the surface of the eyelids, which, in turn, enables their thorough removal. The oil in a concentration of 50% used regularly (once a week) was found to have an excellent eradication effect. Studies by Gao et al. demonstrated effective control of ocular demodicosis using a weekly eyelid peel containing 50% TTO and daily peeling of the eyelids with a tea tree shampoo [103,105,106].

2. Salvia oil has a broad spectrum of applications. In general, salvia was reported to be used in the treatment of eye and skin diseases and oropharyngeal inflammation and possesses mosquito larvicidal properties [9]. Antitumor properties were also reported [107]. According to Sedzikowska et al., 2015 [9], essential oil from the chia plant eliminated *Demodex* spp. effectively and rapidly, while salvia extract was found to be less effective, most likely due to the low content of the essential oils. The authors suggest that the terpenes in essential oils are responsible for the decreasing vitality of *Demodex* mites when applying Salvia preparations. [9]. 

3. Peppermint oil has a broad application range, especially as a flavoring agent in the cosmetic and food industries. It is also proven to exhibit antiseptic properties [108]. According to Thosar et al., 2013 [109], peppermint oil exhibits a significant in-vitro inhibitory effect on common oral pathogens: *Staphylococcus aureus* ATCC 25923, *Enterococcus fecalis* ATCC 29212, *Escherichia coli* ATCC 25922, and *Candida albicans* ATCC 90028 [109]. In the same study, the authors found no significant difference between the antimicrobial activity of peppermint oil and tea tree oil when tested on the above-mentioned pathogens. However, according to Sedzikowska et al., 2015 [9], peppermint oil has a lower inhibitory effect on *Demodex* mites when compared to tea tree oil and salvia oil [9]. The use of peppermint oil as a nanoemulsion (obtained with a high-pressure homogenizer) compared to the peppermint oil in bulk was found to be advantageous, having prolonged the antibacterial activity against *Listeria monocytogenes* Scott A and *Staphylococcus aureus* ATCC 25923, two known foodborne pathogens [105]. This can suggest using nano-emulsions instead of bulk oil to avoid skin irritation or increase the inhibitory effect of the peppermint oil on *Demodex* mites to match the inhibitory effect of more irritating oils, such as tea tree oil. 

4. Castor oil is a natural derivative of the Ricinus communis plant. It is widely used in cosmetic products in the form of emollients (e.g., hair conditioners, creams, and lotions) due to the ease of penetration through human skin, but also, among others, in dressings. It contains fatty acid, which facilitates the regeneration and reconstruction of eyelashes, stimulates their growth, and inhibits the process of hair loss. Castor oil is considered safe and well-tolerated. It has strong properties, including antibacterial, anti-inflammatory, analgesic, antioxidant, wound-healing, and vasoconstrictor aspects. Its important role in replenishing the physiological lipid deficiencies of the tear film, due to ricinolein acid, is indicated. As a result, when applied topically to the eye surface, it is characterized by extended residence time, stability, and an effect on the thickening of the lipid layer of the tear film. Castor oil can be used in the treatment of demodicosis with symptoms of eyelash and eyebrow loss [106]. A promising therapeutic profile was demonstrated in the studies of Muntz et al., 2021 [110], where castor oil was applied topically to patients with marginal blepharitis and significant improvement in symptoms was observed [110]. Hence, the use of castor oil in the treatment of eye surface diseases, including excessive microbial colonization, *Demodex* sp. mites, inflammatory processes, and clinical symptoms of eye dryness and discomfort, is characterized by therapeutic potential [106].

5. Black seed oil—*Nigella sativa* L. (Ranunculaceae), also known as black cumin or black seeds, is a medicinal plant with historical value in traditional medicine used as an essential oil, but also as a paste, powder, or extract, indicated for the treatment of many diseases/conditions. Numerous scientific studies have shown the effectiveness of therapy with the use of black cumin seeds in the treatment of chronic diseases, including inflammation and parasitic infestations, and bacterial, fungal, and viral infections. Nigella sativa oil’s main active component, thymoquinone, plays an important role [111], in addition to others such as thymohydroquinone, thymol, carvacrol, nigellidine, nigellicin, and α-hederin, which are responsible for the pharmacological action and therapeutic benefits. Moreover, black cumin oil has strong antioxidant properties and delays the aging process of the skin. The effectiveness of treatment with Nigella sativa seeds in skin diseases of various etiologies, e.g., skin rashes, acne, and deep wounds, has been reported [112]. It has a significant influence on the condition of the skin, e.g., external use helps to eliminate blackheads and skin imperfections, and additionally strengthens it and makes it more elastic. The oil has a moisturizing, toning, and nourishing effect on the skin. When applied to scars, it reduces their visibility, stimulates the renewal of the epidermis, and visibly tones the skin. It is especially recommended for people with dry, sensitive skin, prone to irritation, but also in the course of infestation caused by *Demodex* spp. mites. It is widely used in the case of skin problems of various etiologies, including acne, blackheads, oily skin, pimples, discoloration, lack of firmness, and signs of aging. The oil softens the skin, soothes irritations, and improves its color. It has been shown to accelerate wound healing and moisturization by accelerating the formation of collagen and the rate of epithelialization. Moreover, it has an antipsoriatic effect [113]. Topical application of the lotion with 10% black cumin oil in animal models significantly alleviated acute and subacute inflammation with a marked inhibition of edema. Based on scientific research, it is suggested that black seed preparations have a beneficial effect on acne vulgaris in humans [114] and in animal models [115], without side effects [112]. In addition, it is pointed out that black-seed therapy could be combined with many conventional chemotherapeutic agents for a synergistic effect. In this way, the effectiveness of the therapy can be optimized, and its toxicity can be lowered. The wide use of Nigella sativa seeds and its oil is emphasized [111].

6. Bergamot oil (BEO) is primarily used as a fragrance oil, especially in aromatherapy. However, in addition to relaxing properties, it is used to treat skin problems and has antiseptic, cooling, toning, and soothing properties. BEO has remarkable biological activity, including pain relief, wound healing, and neuroprotective effects, as well as antimicrobial and anti-inflammatory and even anti-cancer properties [108,116,117]. It supports the nervous system and is also used in alleviating headaches (including migraines). Moreover, BEO is characterized by generally regarded as safe (GRAS) status. Bergamot oil soothes skin irritations and supports the treatment of psoriasis, herpes, and lichen. In addition, it exhibits antibacterial, antiviral, antifungal, and antiparasitic properties [103], which could also be considered when combating demodicosis.

**Table 3:** Treatment.

Available Treatment Options in Demodicosis:			
Antibiotics:	Properties	Treatment	References
Ivermectin	- combats *Demodex* spp. externally and internally - antimicrobial, antiparasitic, antibacterial, and anti-inflammatory activities	- oral treatment of ocular and skin lesions; used alone (e.g., two 200 μg/kg doses, 1 week apart) or in combination with metronidazole (e.g., metronidazole 250 mg 3 times/day for 2 weeks and ivermectin 200 μg/kg doses, 1 week apart); topical, e.g., 1% ivermectin cream once a week for at least 12 weeks	[100,118,119,120]
Metronidazole	- destruction of the pathogen’s DNA structure	- tablets, ointments and gels - topical, e.g., 2% gel - systemic therapy, e.g., 250 mg 3 times/day for 2 weeks	[9,107,121,122,123]
**Alternative treatment:**	**Properties**	**Treatment**	**References**
Tea tree oil	- antiparasitic, antiseptic, antibacterial, antifungal and antiviral - anti-inflammatory and regenerative - accelerates the renewal of the epidermis, process of wound healing, has the potential to heal ulcers - supports the treatment of, e.g., acne, eczema, lichen, and redness	- external use - 50% of tea tree oil (regularly—once a week; 4–6 weeks) for intensive massage of the eyelids and daily hygiene, using a shampoo with tea tree extract - effective control of ocular demodicosis using a weekly eyelid peeling with 50% TTO and daily peeling of the eyelids with a tea tree shampoo	[103,105,106]
Salvia oil	- decreasing vitality of *Demodex* spp.	- topical - eye and skin symptoms relief	[9]
Peppermint oil	- antiseptic	- topical - advantageous use as nanoemulsions	[9,105,108]
Castor oil	- antibacterial, anti-inflammatory, analgesic, antioxidant, wound healing and vasoconstrictor - extended residence time, stability and an effect on the thickening of the lipid layer of the tear film - facilitates the regeneration and reconstruction of eyelashes, stimulates their growth and inhibits the process of hair loss	- eye surface diseases, including *Demodex* spp., inflammatory processes, clinical symptoms of eye dryness and discomfort - form of emollients (e.g., hair conditioners, creams and lotions) - applied topically to the eye surface, demodicosis with symptoms of eyelash and eyebrow loss; marginal blepharitis - considered as safe and well-tolerated	[106,110]
Black seed oil	- accelerate wound healing and moisturization by accelerating the formation of collagen and the rate of epithelialization - strengthens skin and makes it more elastic, moisturizing, toning and nourishing - reduces scar visibility, stimulates the renewal of the epidermis and visibly tones the skin - softens the skin, soothes irritations, improves its color	- treatment of chronic diseases, e.g., inflammation and parasitic infestations, bacterial, fungal and viral infections - skin problems and diseases of various etiologies, e.g., skin rashes, acne and deep wounds, blackheads, oily skin, pimples, discoloration - topical application, external use to eliminate blackheads, skin imperfections - considered as safe	[111,112,113]
Bergamot oil (BEO)	- antiseptic, cooling, toning and soothing, e.g., skin irritations - antibacterial, antiviral, antifungal and antiparasitic properties	- skin problems, wound healing, pain relief - topical	[108,116,117]

## 8. Discussion and Conclusions

There are many considerations about the presence of *Demodex* spp. in humans, its role as a major contributor to human diseases, and the background of developing demodicosis with varied courses of disease. The vast majority of research studies concern adults and the elderly, although recently, more analyses also cover young adults, adolescents, and young children. 

In children and adolescents, *Demodex* spp. is usually low in number and is considered a microorganism, sometimes referred to as a commensal sebaceous unit. However, when it occurs, it may develop a primary eruption or exacerbate facial dermatosis, with the dominance of maculopapular lesions, often on the skin of the face (including the nose) [124]. Zhang et al., 2021 [7] conducted a study in a group of over 1500 children and adolescents aged 3–14 years old, considered healthy (without comorbidities). *Demodex* spp. was found in 12% of respondents with eyelash abnormalities, including trichiasis, cylindrical dandruff, or flaky discharge at the base of the eyelashes [7]. In immunocompromised patients, *Demodex* spp. infestation can cause severe inflammation of the face and eyelids as shown in a study by Damian et al., 2003 [52] in a 6-year-old child. A patient treated for acute lymphoblastic leukemia developed extensive erythema with peeling and skin lesions involving the face and eyelids, bilateral blepharitis, and a lower eyelid chalazion. Testing for demodicosis showed a strong infestation of the hair follicles by *Demodex* spp. Therefore, in severe demodicosis, it is suggested that effective therapy should include the use of, for example, oral ivermectin in combination with topical treatment with permethrin [52]. In the studies of Liang et al., 2010 [33] in patients aged 2.5–11 years with chronic blepharitis and conjunctivitis, an infestation with *Demodex* sp. was shown. They did not respond to conventional treatment only after introducing demodicosis infestation in pediatric blepharitis and conjunctivitis. Patients were treated with therapy lasting several weeks based on the use of 50% tea tree oil (TTO) in the form of eyelid peels or eyelid massage with 5% TTO ointment. In all patients, after a week of TTO treatment, an improvement in eye irritation and inflammation was noted, and the number of *Demodex* sp. decreased. It was also shown that corneal symptoms were essentially resolved within 2 weeks. Furthermore, although one of the patients had a recurrence of demodicosis, the re-introduction of TTO therapy was already successful. Therefore, the application of such a therapeutic model may be beneficial and effective, especially in the case of pediatric patients [33]. 

The condition of the immune system of an infected patient has a crucial impact on the development of the disease and the severity of symptoms [67]. An increased number of mites was observed in people receiving, e.g., steroid drugs, immunomodulating drugs, and chemotherapy, and in individuals infected by HIV or AIDS or dialysis patients [116]. In studies by Kaya et al., 2013 [6] with a group of children, the dependence of infestation with *Demodex* spp. not only with reduced immunity but also with malnutrition was indicated. Approximately one-quarter of malnourished children were infected with *Demodex* spp. A 6-month analysis based on the elimination of poor nutritional status led to the elimination of demodicosis in at least some of the patients [6]. It is therefore suggested that, in addition to being influenced by the state of the immune system, diet may also play an important role in the fight against *Demodex* spp., especially in children. 

It is also worth mentioning the treatment-resistant maculopapular eruptions resistant to antibiotics caused by *Demodex* spp. in the group of children with cancer, and maintenance treatment is necessary in order to prevent delays in the implementation of appropriate therapy [117]. 

In studies by Wu et al., 2019 [125] BKC patients with ocular demodicosis had worse blepharitis and meibomian gland dysfunction and more severe corneal disorders than adults [125]. The importance of differential diagnosis in the case of recurrent or resistant rosacea-like, granulomatous, and perioral acne-like eruptions is emphasized [77]. Although the exact pathogenesis of demodicosis is still discussed, and despite the importance of *Demodex* spp. occurrence in patients in different age groups, there are certain factors that favor the multiplication of these mites and predispose individuals to the development of consequences of varying severity, including local immunodeficiency [76]. The basic diagnostic test of demodicosis is the use of biological material, including standardized skin surface biopsy, skin biopsy, eyelash examination via microscopy, and the use of potassium hydroxide examination [77]. Sometimes, in the case of papulopustular rosacea and demodicosis, clinical diagnosis and diagnostic tests are difficult to carry out due to non-specific symptoms, which are omitted. A study by Forton et al., 2019 [126] emphasizes the importance of other symptoms that may alert dermatologists to perform tests for demodicosis, e.g., the presence of discrete vesicular scales, itching, or inflammation of the scalp follicles [126].

Researchers suggest that clinical changes resulting from infestation with *Demodex* sp. are related to the density of mite individuals, which include blocking of the mouth of the hair follicles. In addition, inflammation and the development of further complications are often associated with the transmission of bacterial infections, e.g., *B. oleronius*, *S. aureus*, or fungal infections carried along with the *Demodex* spp. [127]. In demodicosis and rosacea, the importance of the immune response is emphasized. Studies by Gazi et al., 2019 [121] showed that the reactions of T lymphocytes in the course of infection with *Demodex* spp. and the formation of rosacea have a mutual influence, which can be considered in the development of effective therapies [121]. It is suggested that *Demodex* spp. influence the innate and acquired immune response, and the cellular immunity of the attacked organism plays a key role [122]. Additionally, the influence of *Demodex* spp. mites [31] on the ability to modulate the TLR signaling pathway of the immortalized human sebocyte line and on the immune reactivity of sebaceous cells that secrete interleukin-8 has been suggested [31]. On the other hand, it is also worth taking a closer look at patients with chronic demodicosis, because, as some research studies show, this condition may suggest basic immunodeficiency, e.g., in patients with STAT (signal transducer and activator of transcription) 1 GOF (heterozygous gain-of-function) [123].

In the treatment of demodicosis, various therapies are proposed, based on, among others, medications such as metronidazole, ivermectin, and permethrin, and antibiotics that work against possible bacterial co-infections. The importance of proper diagnosis and the implementation of effective therapy is emphasized, which, especially in the case of dermatoses, disfigures and stigmatizes patients [78]. Currently, the therapeutic standard for demodicosis and the long-term efficacy of the therapies used, which are sometimes associated with skin irritation, have not yet been defined [96]. However, other therapies are sought, among preparations of plant origin, which will be equally therapeutically effective or will support the treatment of demodicosis but with zero or completely minimized side effects and greater tolerance of the skin and eye. The effectiveness of new preparations, such as essential oils, e.g., sage oil, in the treatment of demodicosis, is also significantly influenced by the composition of auxiliary substances, which can both strengthen and weaken their effect in the form of ointments. In studies by Sedzikowska et al., 2015 [9] on the base components of emulsion systems such as liquid paraffin and triglycerides of caprylic and capric acid, longer survival of *Demodex* spp. in 1% of sage oil with paraffin was observed than in the case of triglycerides [9]. TTO with proven effectiveness is one of the most used anti-demodicosis preparations. The research by Ergun et al., 2020 [128] on blepharitis with the use of the TTO gel formula, in the basic form with 3% tea tree essential oil and enriched with vitamins, showed an improvement in the parameters of the eye surface and a reduction of tear cytokines (TNF-α, IL-6, IL-1β) and the numbers of *Demodex* spp. [128]. In addition, based on several scientific studies, it is also indicated that the anti-demodicosis supporting treatment was effective using, e.g., sage oil, peppermint oil [9], castor oil [106], black seed oil [111], chia (Spanish hispanica), and sea buckthorn oil (Hippophae rhamnoides). Studies on certain promising preparations such as clove oil (Oleum Caryophyllorum), orange fruit (Oleum Aurantii), ginger oil (Oleum Zingiberis), cinnamon bark (Oleum Cinnamomi), medicinal alpine rhizome (Rhizoma Alpiniae officinarum), and prickly pear peel (Oleum Opuntia) were found to have a 30 min in vitro killing effect against both *Demodex* species (*D. folliculorum* and *D. brevis*) that cause diseases in humans [129]. In the case of essential oils, attention has to be paid to the potential for irritating the skin or eyes. It is indicated, based on in vivo studies on animal models, that one of the safest is clove oil, which is highly effective in the fight against *Demodex* spp. [104]. Currently, the importance of using alternative therapy, especially with the use of plant-derived preparations in the form of essential oils, is highlighted, although further observations of their effectiveness in vivo will play a key role in the coming years [129]. 

*Demodex* spp. can cause dermatological and ophthalmological problems. Infestation is reported as rather common in adults and the elderly, but more attention has been paid recently to the presence of *Demodex* sp. mites in children, even without comorbidities. The presence of *Demodex* spp. is often asymptomatic and, in many cases, patients struggle with undiagnosed ailments for a long time. Literature reports show that *Demodex* spp. are related to the pathogenesis of numerous dermatoses, including rosacea or demodicosis gravis [130] and common eye dysfunctions. Thus, it is suggested to include, in addition to bacteriological tests, parasitological analysis in dermatological and eye microbiological diagnostics. The main task of therapy is to inhibit the multiplication of parasites, their elimination, and stop the recurrence of invasions. Prevention is based on education on proper hygiene of the skin and the eye area. Antibiotic therapy and essential oils are used in effective therapy, but new efficacious alternative preparations are still being sought. The treatment of patients is a challenge and usually takes a long time, therefore it is important to carefully diagnose and properly select the therapy regimen for the treatment to be successful with reduced side effects, especially for young patients 

## Figures and Tables

**Table 1:** Co-infecting organisms associated with demodicosis.

Microorganism	Symptoms	References
*Staphylococcus* spp. *(S. aureus*, *S. epidermidis)*	blepharitis, conjunctivitis, cutaneous diseases	[24,32,33,34]
*Streptococcus* spp.	blepharitis	[34]
*Bacillus oleronius*	blepharitis, rosacea	[8,33,34]
*Propionibacterium acnes (Cutibacterium acnes)*	blepharitis, acne	[35]
*Corynebacterium* spp.	blepharitis	[36]
Funghi: - *Microsporum canis* (spores) - *Trichophyton* spp.	cutaneous diseases, dermatophytosis, dermatological disorders, pityriasis folliculorum	[21,32,37]

**Table 2:** The most common clinical presentations of demodicosis.

Symptoms of Demodicosis—Summary:	
Skin demodicosis:	
rash, acne, rosacea, unilateral rosacea (unilateral *Demodex* sp. folliculitis), pustules, purulent eruptions, erythema, boils, crusts	[35,83,84]
swelling around skin lesions, burning and itching of the skin, a tickling feeling	[85]
calluses, hyperplasia, peeling of the epidermis (hyperkeratosis), dry skin	[3]
inflammation of follicles	[85,86]
hair loss	[21,83]
blockage of the sebaceous ducts, the formation of blackheads	[83,87,88]
widening of blood vessels leading to skin hyperemia	[21,84]
skin infections caused by bacteria and fungi	[24,34]
Eye demodicosis:	
loss of eyelashes and eyebrows	[21,83]
posterior and/or anterior blepharitis	[24,32,33,34,83]
eye infections caused by bacteria and fungi	[24,34]

## Data Availability

No new data was created during this study.
